# Deciphering the role of trehalose in hindering antithrombin polymerization

**DOI:** 10.1042/BSR20182259

**Published:** 2019-04-05

**Authors:** Asma Naseem, Mohammad Sazzad Khan, Hashim Ali, Irshad Ahmad, Mohamad Aman Jairajpuri

**Affiliations:** 1 Department of Biosciences, Protein Conformation and Enzymology Lab, Jamia Millia Islamia (A Central University), New Delhi-110025, India; 2 Molecular Medicine Laboratory, International Centre for Genetic Engineering and Biotechnology (ICGEB), Trieste 34149, Italy

**Keywords:** antithrombin, chemical chaperones, polymerization, serpin, thrombosis, trehalose

## Abstract

Serine protease inhibitors (serpins) family have a complex mechanism of inhibition that requires a large scale conformational change. Antithrombin (AT), a member of serpin superfamily serves as a key regulator of the blood coagulation cascade, deficiency of which leads to thrombosis. In recent years, a handful of studies have identified small compounds that retard serpin polymerization but abrogated the normal activity. Here, we screened small molecules to find potential leads that can reduce AT polymer formation. We identified simple sugar molecules that successfully blocked polymer formation without a significant loss of normal activity of AT under specific buffer and temperature conditions. Of these, trehalose proved to be most promising as it showed a marked decrease in the bead like polymeric structures of AT shown by electron microscopic analysis. A circular dichroism (CD) analysis indicated alteration in the secondary structure profile and an increased thermal stability of AT in the presence of trehalose. Guanidine hydrochloride (GdnHCl)-based unfolding studies of AT show the formation of a different intermediate in the presence of trehalose. A time-dependent fluorescence study using 1,1′-bi(4-anilino)naphthalene-5,5′-disulfonic acid (Bis-ANS) shows that trehalose affects the initial conformational change step in transition from native to polymer state through its binding to exposed hydrophobic residues on AT thus making AT less polymerogenic. In conclusion, trehalose holds promise by acting as an initial scaffold that can be modified to design similar compounds with polymer retarding propensity.

## Introduction

Antithrombin (AT) is the main regulator of the blood coagulation cascade and acts by inhibiting various proteases like thrombin, factor IXa, factor Xa and factor XIa [1,2]. Point mutations in AT can lead to its oligomerization and polymer formation which decreases its levels in plasma and might result in thrombosis [3–7]. AT belongs to the serine protease inhibitors (serpin) superfamily, members of which have the same structure and mechanism of inhibition. A serpin is composed of three β-sheets (A–C), 7–9 α-helices (hA-hI) and a mobile reactive center loop (RCL) which is exposed [8,9]. This loop presents a peptide sequence as a pseudo-substrate for the target proteinase that is cleaved after docking with the enzyme [10]. A large conformational change then drags the protease from the top to the opposite pole that leads to the formation of a thermodynamically stable serpin-protease complex [11,12]. However, in many serpins including AT, this inhibition mechanism renders them susceptible to form inactive ordered polymers by introducing point mutations that allow the entry of RCL of one molecule into β-sheet A of another [13–15]. Mutation resulting in polymer formation in α-1antitrypsin (AAT), neuroserpin (NEU), AT, C1 inhibitor, antichymotrypsin (ACT) and heparin cofactor II (HCF-II) can lead to pathological states like cirrhosis, emphysema, dementia, thrombosis and angioedema [16].

Several RCL-based peptides have been used to block polymerization which acts by annealing to the β-sheet A, but they also abrogates the normal function of serpin [17,18]. In antitrypsin, hydrophobic pocket filling using space filling variant dramatically reduced the polymer formation [19]. Use of Glycerol, phenyl butyric acid and trimethylamine N-oxide (TMAO) has been shown as another pharmacological strategy that ameliorated liver cirrhosis [20,21]. Alcohols and sugar molecules like glycerol, erythritol and trehalose were effective in reducing the rate of polymerization of wild-type and mutant NEU [22]. Small molecules can have advantage of being directly administered to prevent *in situ* polymerization and reduce cell toxicity [23]. Effective leads that can act at low concentration are desirable; however, despite advances in understanding the role of these compounds in serpin polymerization, interaction of these compounds, modulation of conformation and inhibitory mechanism is not clearly established. Here, we report an *in vitro* screening of small molecules belonging to the sugar, amino acids and methlyamines that lead to the identification of few potential leads as they successfully retarded AT polymer formation assessed by Native-PAGE. Among them, the disaccharide trehalose proved to be most promising as it was effective at the lowest concentration of 1 M. Furthermore, the kinetic and thermal stability data of trehalose was the best among all the other leads. In view of this, we refined our screening and performed a comprehensive circular dichroism (CD)- and fluorescence-based structural study of AT with trehalose. The ensuing results indicate that presence of trehalose at concentrations that reduce polymer formation can induce changes in the overall stability, secondary structure and hydrophobic profile of AT resulting in reduction of ‘beads on a string like AT polymer’. It is hypothesized that trehalose-based structural analogs could be developed to be more effective at lower concentrations and act as potential therapeutics to treat polymerization-based serpinopathies.

## Materials and methods

### Materials

Hi-Trap heparin high affinity columns were purchased from GE Biosciences and the integrated protein purification system was from Biorad. Amicon Ultra-15 centrifugal filters were from Millipore. Human thrombin (IIa) and S-2238 (H-D-Phenylalanyl-L-pipecolyl-L-arginine-p-nitroaniline dihydrochloride) were from American Diagnostic. Ultrapure guanidine hydrochloride (GdnHCl) was purchased from MP Biomedicals. 1,1′-bi(4-anilino)naphthalene-5,5′-disulfonic acid (Bis-ANS) was from Sigma–Aldrich. Small molecules used in the study were purchased from Merck.

### Preparation of buffers, denaturant (GdnHCl) and fluorophore (Bis-ANS)

For most experiments, PE buffer was used (20 mM sodium phosphate containing 0.1 mM EDTA, pH 7.4). However, for AT-thrombin activity measurements, 100 mM NaCl and 0.1% of PEG6000 were also added to PE buffer in order to make it suitable for 96-well plate measurements. After pH adjustment, all the buffers were filtered with 0.22-μm Millipore syringe filters and stored at 4°C for use. For preparing GdnHCl stock solution, an appropriate amount of GdnHCl was weighed and dissolved in PE buffer. After pH adjustment, solution was filtered and the concentration was determined from the value of difference between the refractive indices of the denaturant and the buffer solution at room temperature using the following equation [24]:

(1) C=57.147(ΔN)+38.68(ΔN)2−91.6(ΔN)3

where C is the concentration of GdnHCl in moles per liter and ΔN is the difference between the refractive indices of the denaturant and buffer solutions. In the present study, hydrophobic aromatic fluorescent dye 4-4′dianilino-1,1′-binaphthyl-5,5′- disulfonic acid (bis-ANS) was chosen. Appropriate amount of bis-ANS was precisely weighed and dissolved in PE buffer. The concentration of bis-ANS was determined spectrophotometrically using its molar extinction coefficient [25].

### Purification of AT from human plasma and induction of polymerization

AT Purification from human plasma was achieved by using Hi-Trap heparin affinity column which was eluted with a 0.15–2.50 M NaCl gradient as described earlier [26]. Concentration of purified AT was determined by measurement of UV absorbance at 280 nm using molar extinction coefficient of plasma AT [27]. Polymer formation of AT was induced by heating AT under specific buffer and pH conditions. 100 µg ml^−1^ of native AT in a total of 1 ml was incubated at 60°C in 50 mM Tris buffer and 50 mM KCL, pH 7.4 in the absence and presence of small molecules (listed in Supplementary Table S1) at different time intervals. Samples were removed at indicated times, snap frozen and stored at −70°C for further analysis. Native-PAGE was done to visualize the high molecular weight polymer bands of AT.

### Thrombin inhibition kinetics by AT

Kinetics of thrombin inhibition by AT in the presence of lead molecules were determined by taking AT in three different concentrations (100, 200 and 300 nM) in PNE-PEG buffer. These were made to react with thrombin (10 nM) in the presence of trehalose (1.0 M), sorbitol (1.5 M), mannose (1.5 M each), serine (1.25 M) and TMAO (1.0M) at given time-points in a 96-well plate. Absorbance was taken at 405 nm after the addition of thrombin substrate S-2238 (0.15 mM). Stoichiometries of thrombin inhibition (SI) by AT in the absence and presence of lead molecules were determined as described previously [26]. *k_assoc_* rates were quantified from the plots obtained. Appropriate thrombin and S2238 controls/blanks with small molecules in the absence of protein were taken.

### Transmission electron microscope analysis

AT polymer formation was assessed in the absence and presence of sorbitol and trehalose for 0 and 90 min. Polymers were formed by heating AT at 60°C for 90 min in PE buffer at pH 7.4. Aliquots were withdrawn at 0 and 90 min and the reaction was quenched by placing the sample on ice. Copper/formvar grids of 300 mesh were prepared for the samples that were stained negatively with 1.5% (w/v) uranyl acetate and viewed with a magnification of upt o ×50000 with transmission electron microscope (TEM) (FEI Morgagni 268 D with digital camera Image 268 D).

### Fluorescence measurements

Fluorescence spectra were recorded on a JASCO 6300 spectrofluorimeter using a 1-cm quartz cell. A slit width of 5 nm was used for both excitation and emission wavelengths. For the bis-ANS fluorescence in probe–protein binding experiments, samples were excited at wavelength of 385 nm and the emission spectra were recorded from 390 to 600 nm using a scan speed of 100 nm/min in 1-nm steps and an integration time of 5 s. The concentration of AT was 1 µM and the molar ratio of AT to bis-ANS was 1:10. Data were corrected by subtracting the buffer contribution.

### Circular dichroism measurements

CD spectra were obtained using an Applied Photophysics spectropolarimeter at 25°C with 1 nm/10 s signal. The far-UV CD spectra (200–260 nm) were recorded using a 1 mm path length cell. For the near-UV CD (260–310 nm) measurements, a 5 mm path length cuvette was used. Far-UV and Near-UV CD spectra of AT in the absence and presence of effective concentrations of lead molecules were acquired at 25 ± 0.1°C. Each spectrum was corrected for blank contribution. Thermal unfolding was performed using a heating rate of 60°C/h, and the changes in secondary structure with temperature were measured by monitoring the CD signal at 222 nm. Melting points (Tm) were calculated as described [20]. Protein concentration was 3 μM and that of trehalose was 1 M.

Raw CD data were converted into the mean residue ellipticity, MRE (deg.cm^2^ dmol^−1^) at wavelength λ using the relation:

(2) $$\text{MRE}=\frac{\left({\text{M}}_{\text{o}}*\text{θ}\right)}{10*1*\text{c}}$$

where (*θ*) is the observed ellipticity in millidegrees at wavelength λ, *M_o_* is the mean residue weight of the protein, *c* is the protein concentration in milligrams per milliliter, and *l* is the path length of the cell in centimeters.

### GdnHCl-induced unfolding transition

Unfolding as a function of GdnHCl concentration was monitored by CD and fluorescence spectroscopy. To AT solution (500 nM in PE buffer, pH 7.4) in the absence and presence of 1 M trehalose, aliquots of 8.2 M GdnHCl were added in order to obtain the desired denaturant concentration (0–6 M). These samples were then used for fluorescence and CD measurements.

### Statistical analysis

Data for temperature dependence bis-ANS experiment were analyzed with two-way ANOVA (bonferroni post-tests) while AT-thrombin activity data were analyzed using linear regression application of GraphPad PRISM software (version 5, San Diego, CA, U.S.A.).

## Results

### Small molecules retards AT polymerization

AT was purified from human plasma as shown in Supplementary Figure S1 and conditions were provided to induce polymer formation. Supplemenatry Figure S2A shows behavior of native AT under polymerization condition where an increased polymerization is seen upon longer incubation. Most of the molecules used in the *in vitro* screening (Supplementary Table S1) had no effect on AT polymer formation (data not shown). However, five of them namely trehalose, sorbitol, mannose, serine and TMAO successfully blocked long chain polymers of AT in a concentration dependent manner as seen on native-PAGE gels (Supplementary Figure S2B–N). The effective concentrations were 1, 1.5, 1.5, 1.25 and 1M for trehalose, sorbitol, mannose, serine and TMAO, respectively. These effective concentrations respective for each molecule were then carried forward in further experiments.

### AT activity and stability in the presence of small molecules

**Figure 1:**
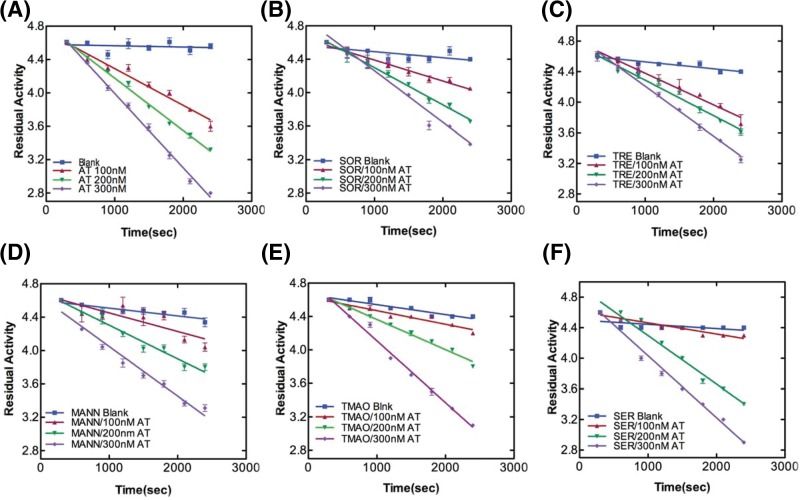
Rate of thrombin inhibition by AT in the absence and presence of small molecules Kinetics of thrombin inhibition by AT were determined by reacting AT in three different concentrations (100, 200 and 300 nM) with thrombin (30 nM) in the **(A)** absence and presence of (**B**) 1.5 M sorbitol, (**C**) 1 M trehalose, (**D**) 1.5 M mannose, (**E**) 1 M TMAO and (**F**) 1.25 M serine at given time-points in a 96-well plate. Absorbance was taken at 405 nm after the addition of thrombin substrate S-2238 (0.15 mM). Appropriate thrombin and S2238 controls/blanks with small molecules in the absence of protein were taken. Measurements were carried out at least three times and data were analyzed with linear regression function of GraphPad Prism v5.0.

**Figure 2:**
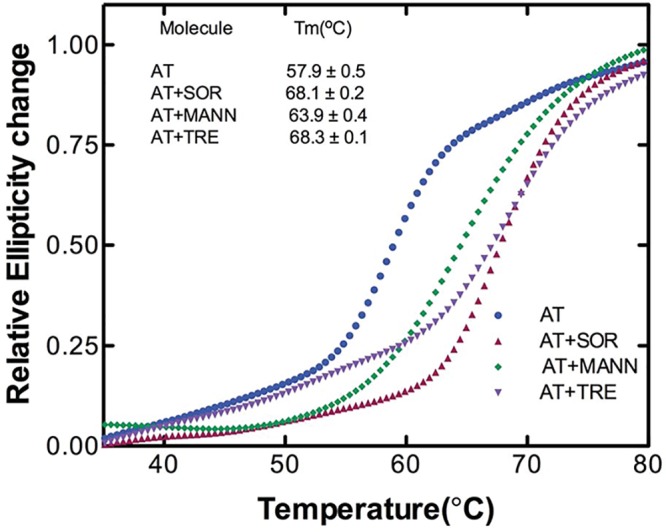
Stability of AT in the absence and presence of small molecules Relative ellipticity change at 222 nm is plotted against temperature over the range of 35–80°C of 3 µM of AT in the absence and presence of 1 M trehalose, 1.5 M sorbitol and 1.5 M mannose as described in methods. Relative ellipticities were calculated by using the relation [θ_obs_− θ_min_]/[θ_max −_θ_min_] [38] where θ_min_ and θ_max_ were fitted values of ellipticity at the lowest and highest temperatures used in the study, respectively, and θ_obs_ is the observed ellipticity at temperature T. Each curve is an average of three experiments.

The rates of thrombin inhibition by AT were determined from continuous assays using the change in absorbance at 405 nm resulting from hydrolysis of chromogenic substrate. Figure 1A–F shows the progressive thrombin inhibition plots for AT in the absence and presence of lead molecules in which residual activity is plotted against time. The slopes gave us the pseudo-first order *(k_obs_)* and second-order rate constants (*k_app_*). SI experiments showed a value of 1 in the absence and an increased stoichiometry (>1 but <2) in the presence of sugar molecules suggesting a slowing down of RCL insertion (Supplementary Figure S3). From the values of *k_app_* and SI, overall association rate constants, *k_assoc_* were calculated (Table 1). Antithrombin RCL inserts between strand 3A and 5A of β-sheet A as strand 4A after protease binding and cleavage. Trehalose, sorbitol and mannose have been shown to bind to the upper shutter region around strand 6A [28]. Taken together, the results show a slight slowing down of loop insertion in the presence of lead molecules that concomitantly decreases A-sheet motility thereby diminishing the tendency for intermolecular loop-sheet interactions based AT polymerization. Next, we measured thermal stability of AT in the absence and presence of lead sugar molecules (Figure 2). We observed a Tm of 57.9°C for AT consistent with previous reports [5,20]. A 10°C increase in Tm in the presence of 1.5 M sorbitol and 1 M trehalose and a 6°C increase in Tm in the presence of 1.5 M mannose was observed. Increase in the Tm is indicative of increase in the overall stability of native AT. It has been shown that trehalose has the propensity to bind to the native state at elevated temperatures thus providing a more compatible environment that protects proteins from heat inactivation [29]. This quality of preferential hydration in trehalose (and other sugars) led to an attainment of stability of AT during the course of denaturation. It seems sorbitol and to a lesser extent mannose also led to an increased stability of AT in a similar manner.

**Table 1:** Thrombin inhibition kinetics by AT.

Molecule	*_k(app_*_)_*10^−3^ (M^−1^s^−1^)	SI	k_(assoc)_*10^−3^ (M^−1^s^−1^)
AT	3.3 ± 0.1	1.0 ± 0.1	3.3 ± 0.1
AT + SOR	2.6 ± 0.2	1.8 ± 0.1	4.7 ± 0.1
AT + MANN	2.7 ± 0.2	1.7 ± 0.1	4.6 ± 0.2
AT + TRE	2.9 ± 0.1	1.5 ± 0.2	4.4 ± 0.1
AT + SER	2.5 ± 0.2	2.1 ± 0.1	5.3 ± 0.1
AT + TMAO	2.3 ± 0.2	2.7 ± 0.2	6.2 ± 0.2

Apparent second-order rate constant (*k_app_)*, inhibition stoichiometries (SI) and second-order association rate constants (*k_assoc_)* of thrombin inhibition by AT in the absence and presence of small molecules. Observed pseudo-first-order rate constants, *k_obs_* were obtained from the negative slope of a plot of residual enzyme activity versus time (of thrombin and AT co-incubation). *k_app_* were calculated by dividing *k_obs_* by molar concentration of AT. k_assoc=_ k_app_* S.I. Mean ± S.E.M. of three independent experiments is shown. Abbreviations: MANN, mannose; SER, serine; SOR, sorbitol; TMAO, trimethylamine N-oxide; TRE, trehalose.

### Trehalose and sorbitol leads to a reduction in the size and shape of AT polymers

**Figure 3:**
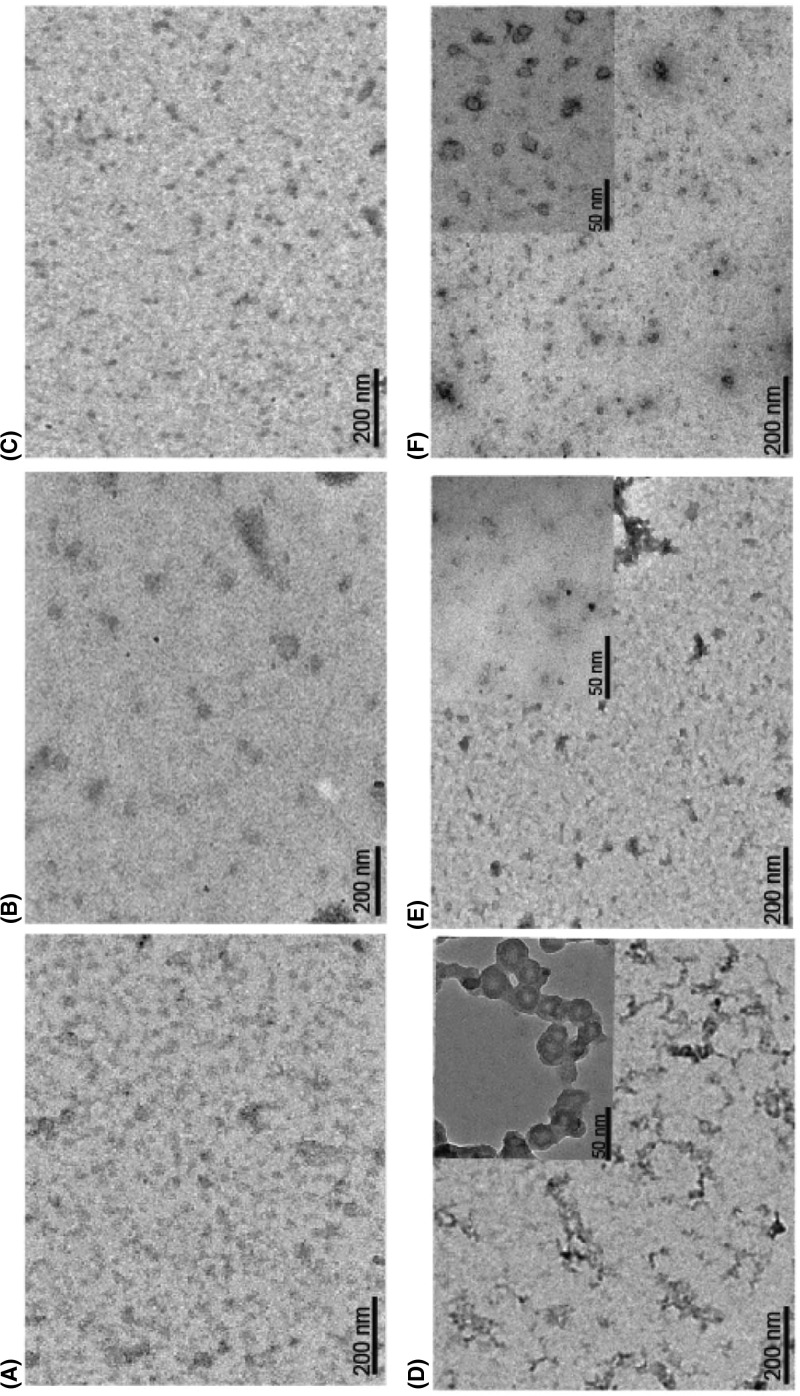
Electron micrographs showing reduction in the size and shape of AT polymers by sorbitol and trehalose AT Polymers were formed by heating 10 µM of the protein at 60°C for 90 min. Samples were stained negatively with 1.5% (w/v) uranyl acetate, and viewed with a magnification of up to ×50000. AT incubated at (**A**) 0 min, (**D**) 90 min; AT incubated in the presence of 1.5 M sorbitol at (**B**) 0 min and (**E**) 90 min and in the presence of 1 M trehalose at (**C**) 0 min and (**F**) 90 min. Insets in D–F shows enlarged view of polymers.

Members of serpin superfamily unlike amyloid fibrils are known to form bead-like polymers that accumulate inside the cells and result in pathological states like liver cirrhosis [30] and dementia [31]. We examined the nature of AT molecule in the absence and presence of 1.5 M sorbitol and 1 M trehalose using TEM. Figure 3A shows scattered-AT monomers at native state that upon incubation at 60°C for 90 min took the shape of bead-like polymers (Figure 3D). Treatment with 1.5 M sorbitol (Figure 3E) and 1 M trehalose (Figure 3F) led to a reduction of these polymers. The results confirmed that the high molecular weight polymers of AT seen on native-PAGE (Supplementary Figure S2A) were reduced in the presence of these molecules, albeit the effect of sorbitol was not as strong as trehalose. It implies that if trehalose shields AT, the insertion of RCL will be hampered due to compactness of trehalose bound AT. The effect can arise either due to trehalose-induced conformational change reducing the exposure of hydrophobic surfaces or due to trehalose directly interacting with hydrophobic surface creating hinderance for RCL insertion from other AT molecule to form polymer. In conclusion, TEM analysis showed generation of bead like AT polymers upon heat incubation owing to significant structural changes with newly exposed hydrophobic surfaces. These polymers get truncated in the presence of trehalose which shields AT from intermolecular interaction by reducing its hydrophobicity thus limiting polymerization since hydrophobicity is a property of serpin polymers [32].

### Effect of trehalose on native, intermediate and unfolded state of AT

**Figure 4:**
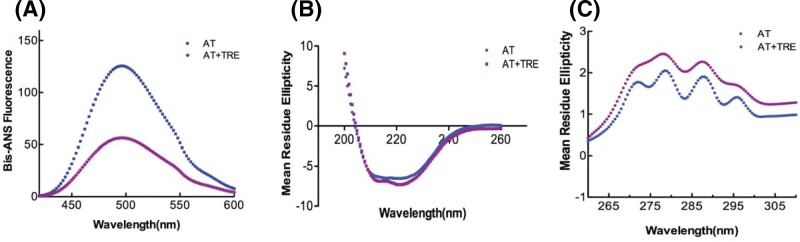
Effect of trehalose on native state of AT (**A**) Bis-ANS fluorescence, (**B**) Far-UV CD and (**C**) Near-UV CD spectra of native AT in the absence and presence of 1 M trehalose. Experimental details are mentioned in Materials and methods section. All measurements were carried out at 25°C in 20 mM phosphate buffer containing 100 mM NaCl and 0.1% EDTA (PNE buffer). The concentration of protein was 1–20 µM and that of trehalose was 1 M. All the data were corrected for buffer effect. Each curve represents an average of three (for Bis-ANS and Far-UV CD) and two (for near-UV CD) independent experiments.

To fully explore the role of trehalose in hindering AT polymerization, it was important to understand its effect on the various states of AT that prevails in solution. For this, we studied the effect of 1 M trehalose on different states of AT through CD and fluorometric analysis. Bis-ANS binds to proteins and can provide information about the relative exposure of hydrophobic surfaces [33]. Figure 4A shows the Bis-ANS emission spectra of native AT in the absence and presence of 1 M trehalose. With trehalose, >2-fold decrease in the emission intensity of AT was observed indicating a shielding of exposed hydrophobic surfaces of AT on account of trehalose binding. Figure 4B shows the Far-UV CD spectra of AT indicating an increase in the α-helical content in the presence of 1 M trehalose. Figure 4C shows the near-UV CD spectra under similar conditions, where we observed an absence of Phe peak and a shift in the tyrosine and tryptophan peaks to 276 and 287 nm, respectively, indicating an overall change in the tertiary structure of AT. The data indicates that in the presence of polymer reducing concentration of trehalose (1 M), increased compactness of AT reduces the overall exposed hydrophobic surface by increasing the α-helical content and altering the tertiary structure.

**Figure 5:**
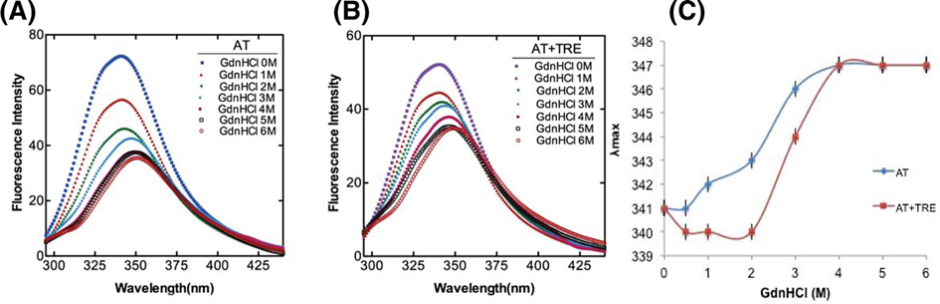
GdnHCl-based denaturation profile of AT GdnHCl (0–6 M)-induced unfolding transition of native AT was measured by the change in the fluorescence signal. Representative tryptophan fluorescence spectra of AT were recorded in the (**A**) absence and (**B**) presence of 1 M trehalose. All the measurements were carried out with 500 nM of AT incubated with GdnHCl (0–6 M) for 2 h prior to fluorescence measurements at 25°C in PNE buffer. An excitation wavelength of 280 nm was used and the scan was recorded from 295 to 440 nm. (**C**) Emission maxima plot extrapolated from the chemical denaturation profile of AT by GdnHCl (0–6 M) monitored by intrinsic tryptophan fluorescence in the absence and presence of 1 M trehalose. Each plot is an average of measurements carried out at least three times.

**Figure 6:**
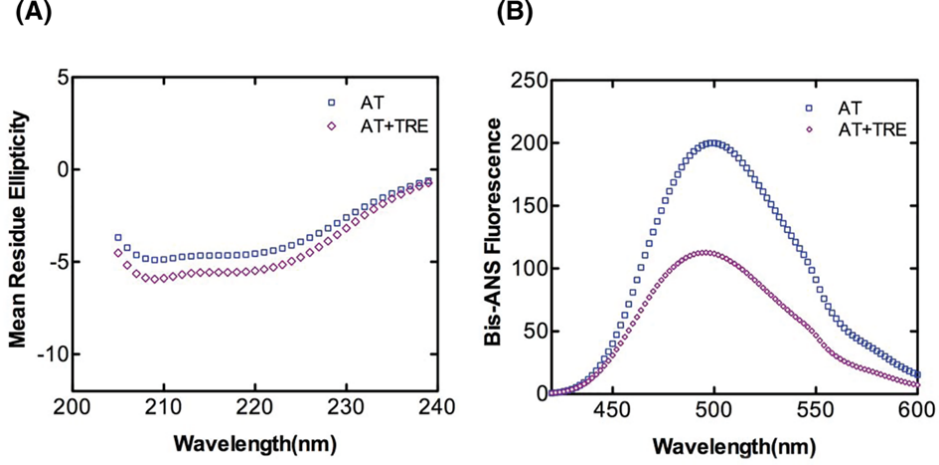
Effect of trehalose on intermediate state of AT (**A**) Far-UV CD spectra and (**B**) Bis-ANS fluorescence of AT in the absence and presence of 1 M trehalose was done to observe conformation of intermediate state. AT was incubated with 2 M GdnHCl for 2 h in the absence and presence of 1 M trehalose prior to CD and Fluorescence analysis. The concentration of AT was 1 µM and the molar ratio of AT to bis-ANS was 1:10. Data were corrected by subtracting the buffer contribution. All data points in each plot were obtained from the average value of atleast three independent experiments.

Next, we forwarded our study to unfolded states of AT and used the chaotrope GdnHCl to pursue this objective. Figure 5 shows fluorescence spectra curves of AT in the absence (panel A) and presence of 1 M trehalose (panel B). We observed that GdnHCl mediated unfolding of native AT showed a decrease in emission intensity with a significant red shift (Figure 5A). Incubation with 1 M trehalose also showed a similar pattern of reduced fluorescence emission intensity with a red shift indicating a change in the microenvironment of the tryptophan residues (Figure 5B). Figure 5C shows the emission maxima plot of AT in the absence and presence of 1 M trehalose with increasing GdnHCl concentration. As can be seen from the plot, a peak centered at 341 nm characterized native AT. The fluorescence markedly changes when the protein undergoes unfolding, with a shift in the emission maximum to 343 and 347 nm at 2 and 6 M GdnHCl concentrations, respectively. Incubation with 1 M trehalose did not have much effect on emission maxima of native AT (0 M GdnHCl) and unfolded AT (6 M GdnHCl); however, there was a return of emission maxima to 340 nm at 2 M GdnHCl. This blue shift of the maximum of fluorescence emission at 2 M suggests that most of the tryptophan residues have recovered the environment close to the one they have in the native state. Further, from the plot of AT, we observed two well resolved phases of unfolding: one from 0 to 2 M and another from 3 to 6 M GdnHCl concentration indicating a three state transformation to unfolded state with an intermediate around 2 M GdnHCl. The results agreed with the three state transformation to unfolded state of AT shown previously [34]. In the presence of 1 M trehalose, however, we observed the formation of a different intermediate structure with native AT like characteristics. To further investigate the impact of trehalose on intermediate state of AT, we acquired far-UV CD spectra of AT as shown in Figure 6A. It can be seen that in the presence of 2 M GdnHCl, trehalose increases the α-helical content of AT by approximately 6% (Supplementary Table S2). Although the effect is small, the trend agrees with the interpretation that trehalose affects intermediate state of AT. A fluorescence emission spectra in the absence and presence of 1 M trehalose along with bis-ANS and 2 M GdnHCl is shown in Figure 6B. The results show a massive increase in emission intensity of AT folding intermediate as compared with native control (see also Figure 4A). Further, incubation with 1 M trehalose shows a reduction in the exposure of hydrophobic surface of the intermediate as compared with the 2 M GdnHCl incubated AT. The fluorescent intensity of AT intermediate in the absence of trehalose is more than the fluorescent intensity for the native AT (Figure 4A), implying that the overall surface hydrophobicity of AT intermediate is more than the native and that trehalose decreases both of them.

### Time dependence of polymerization

**Figure 7:**
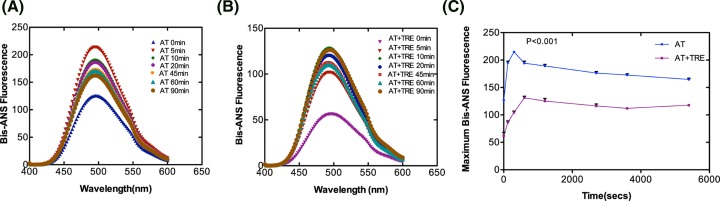
Time dependence of AT studied by bis-ANS fluorescence Time course of AT polymerization in the (**A**) absence and (**B**) presence of 1 M trehalose. 2 µM AT in a total of 1 ml in PNE buffer was incubated at 60°C at indicated times in the absence and presence of 1 M trehalose. Samples were removed post-incubation and increase in fluorescence was measured at 485 nm after excitation of samples at 385 nm in bis-ANS. (**C**) Fluoresence intensity versus time plot. Average points from three separate experiments each with five scans were used to plot the graphs. Data were analyzed with two-way ANOVA (bonferroni post tests) and a *P*-value ≤0.01 was considered significant.

Bis-ANS binding at various time points under the polymerization condition was done to assess the relative exposure of hydrophobic surfaces during the course of polymer formation. Figure 7 shows the binding of bis-ANS to AT during polymerization in the absence (panel A) and presence of 1 M trehalose (panel B). The result in Figure 7A shows a rapid increase in emission intensity of AT on account of bis-ANS binding followed by a decrease in emission intensity with 90 min being the point of lowest intensity. The data show a gradual increase in exposure of hydrophobic core, which is an indication of conformational deformation due to partial unfolding that stabilizes after 10 min. However, in the presence of 1 M trehalose (Figure 7B), overall fluorescence intensity is decreased throughout the course of polymerization. Data points from these spectra were also plotted in fluorescence intensity/time graph as shown in Figure 7C where we can appreciate the phase formation during the course of polymerization. We observed two phases that can be deciphered as an initial rapid conformational deformation phase due to partial unfolding and a stabilization phase indicating polymerization. Of note, there is a decrease in emission intensity of AT in the presence of trehalose which implies that trehalose reduces the hydrophobic surface (which is concurrent with a decrease in emission intensity) and also slows down the rate of initial conformational change step during polymerization. The results show that a partially denatured AT when stabilized with 1 M trehalose resists the transition to polymerized form through reduction in hydrophobic core and probably an increase in hydration; both these factors contribute to maintain AT native state.

## Discussion

The process of serpin polymerization is of acute biomedical interest given the recognition that several devastating pathologies including thrombosis are etiologically correlated to serpin polymers. The underlying reason for most of the serpinopathies is the sophisticated mechanism of serpin inhibition that involves a large conformational change which makes it prone to conformational deformation based diseases [15,16]. Reversing the accumulation of polymerized serpin to decrease cytotoxicity is central to the design of novel strategies for its cure. To date, very few systematic studies have been performed that found compounds to ameliorate serpinopathies. In α1-antitrypsin, polymerization leads to both emphysema and severe liver disease and introduction of a bulkier group by mutation within a cavity or incubation with chemical chaperone like TMAO (1.5–3.0 M) were shown to retard polymer formation [21,35]. It is important to rationally design compounds that hinders polymerization but has minimum cross reactivity, maintains activity and does not contribute to cellular toxicity. In the present work, we screened small molecules (Supplementary Table S1) to identify *bona fide “*hits” that could prevent AT polymerization. We performed polymerization experiments of AT in the presence of varying concentrations of the molecules and analyzed them through Native-PAGE. Out of all the screened molecules, we succeeded in identifying trehalose, sorbitol, Mann, serine and TMAO at given concentrations that completely blocked AT polymerization (Supplementary Figure S2). We then analyzed their effect on normal AT function to inhibit thrombin in order to further filter the hits. We observed a slight increase in *k_assoc_* values in the presence of lead molecules; however, among them the measures of activity for trehalose were the closest to normal AT values (Figure 1 and Table 1). This suggests that addition of trehalose to the AT-thrombin reaction mix does not alter the basic mechanism or efficiency of proteinase inhibition significantly (Supplementary Figure S3 and Table 1). Ligand–protein interactions usually modifies the midpoint of the melting curve (Tm). Thermal stability study of AT in the presence of trehalose resulted in a rise of 10°C in Tm as compared with AT alone (Figure 2). This increase was attributed to the changes in AT conformational flexibility induced by trehalose binding as evidenced in Figure 4 where we observed an increase in the secondary structure and overall change in the tertiary structure of AT upon incubation with trehalose (Figure 4 and Supplementary Table S3) Increase in Tm was also observed for sorbitol and mannose by 10 and 6°C, respectively; however, their effect on AT structure was not as remarkable as that of trehalose (data not shown). Concerning the morphology of polymeric species, TEM analysis was performed on heat-induced polymers of AT in the absence and presence of trehalose. The micrographs showed generation of bead like AT polymers that looked longer and rigid. Further, it was observed that the polymers got truncated and appeared less stiff upon incubation with trehalose which seems to shield AT from protein–protein interaction (Figure 3). The effect of sorbitol was also monitored but it was marginal as compared with trehalose.

Serpin is proposed to undergo polymerization using two distinct steps, the native state intermediate undergoes a change to a polymeric intermediate that self-associates to a dimer to form long chain polymer [13,36]. AT folds into a native state using a molten globule type intermediate which is disturbed in variants that undergo polymerization [34]. Chemical denaturation studies using GdnHCl with native AT showed a three state transformation to unfolded state with an intermediate around 2 M GdnHCl. Native AT showed an emission maximum of 340 nm with a marked change in fluorescence in the intermediate (2 M GnHCl) and unfolded state (6 M GdnHCl) and a shift in the emission maxima to 343 and 347 nm, respectively. However, after the addition of 1 M trehalose to 2 M GdnHCl-denatured AT, the emission maximum was blue shifted to 340  nm suggesting that the presence of trehalose bring in some conformational changes in AT that kept it in a native like conformation. Additionally, there was no notable effect of trehalose on completely unfolded AT as the emission maxima remained the same (Figure 5). Effect of trehalose on AT intermediate showed that when treated with trehalose, intermediate state had a native like secondary fold (Figure 6). It indicates that trehalose forces the intermediate of AT to fold into a native like conformation by increasing its α-helical content thereby making it partially folded and decreasing its hydrophobicity (as observed by a drop in bis-ANS fluorescence). Bis-ANS binding experiments with AT folding intermediate also showed considerable reduction in the exposure of the hydrophobic surface in the presence of trehalose which was concurrent with an increased α-helical content (Figure 6). For AT-bisANS binding at various time points under the polymerization condition, we observed an initial increase in emission intensity indicating the initial conformational change followed by polymerization phase with an increase in incubation time and a drop in fluorescence intensity owing to polymerization induced conformational change in AT (Figure 7A). After the addition of trehalose to the reaction mix, we observed a sharp drop in fluorescence intensity at native state and during the course of polymerization. This implies that through its binding to exposed areas rich in hydrophobic residues on AT, trehalose reduces the hydrophobicity of AT making it less polymerogenic (Figure 7B).

From our *in silico* analysis of AT-trehalose interactions, we observed that AT interacts with trehalose at the interface between the strand 6A and strand 5A, very near to the region where the RCL inserts as s4A and made important hydrogen and hydrophobic interactions [28]. Taken together, our results have shown that effect of trehalose on the polymerization of AT is based on the loop sheet model. Contrarily, if we assume a set up to study the role of trehalose in combating AT polymerization (caused by domain swapping), it is most likely that trehalose will affect the intermediate M* state. This is because M* state is shown to be a highly hydrophobic moiety sensitive to polymerization/aggregation [13] and our bis-ANS data have shown that trehalose has a very high tendency to bind to hydrophobic regions on AT and thereby diminishes the chances of AT polymerization (Figures 4A, 6B and 7).

Preferential hydration is the exclusion of co-solvents like sugar from native state and acts as the main driving force for protein stabilization [37]. The present study clearly demonstrates that trehalose not just rescues AT from temperature induced polymerization but at the same time is also helpful in the retention of its inhibitory activity (Supplementary Figure S4). Massive decrease in hydrated hydrophobic surfaces on incubation with trehalose and an increase in the stability of the native state points to the importance of preferential hydration in controlling protein–protein interaction. It is concluded that trehalose can disturb the hydration around AT affecting the solvation energy. Given the fact that trehalose acts as a universal protein stabilizer that is effectively used to increase the stability of many of the industrial and therapeutic enzymes, it will be intriguing to test its efficacy in controlling thrombosis and other diverse serpinopathies. Further, it is also plausible to use analog of trehalose in controlling polymerization rates for minimizing the effective depolymerization concentration (Supplementary Figure S5).

## Supporting information

**Figure S1:** Purification of AT from human plasma. Elution profile of purified AT is shown. (A-C) SDS-PAGE of AT purified using a salt gradient of NaCl (20 mM phosphate buffer containing 100mM NaCl, 0.1mM EDTA, pH 7.4 and ionic strength 0.15). Elution of protein started from 0.15M NaCl upto 2.5M NaCl. (A) Lanes showing the fractions collected from 0.5M upto 1M NaCl gradient run in doublets as indicated. (B) Fractions collected from 1.25M upto 1.75M are shown as in A. (C) Fractions from 2M upto 2.5M NaCl are shown as in panels A and B. Fractions containing 0.15-0.5 M gradients were treated as wash. Fractions showing single bands of purified protein were pooled, desalted and used in the study. M denotes pure AT run as marker. Lanes denotes different concentrations of NaCl as indicated. (D) Absorbance profile of the eluted fractions is shown. Each fraction was eluted for 3 minutes and absorbance was read at 280nm.

**Figure S2:** Concentration dependence of small molecules that successfully retarded AT polymerization. Polymers of AT were prepared by heating 100μg ml^-1^ of native AT in total of 1ml at 60°C in 50 mM Tris and 50 mM KCL buffer, pH 7.4 in the absence (A) and presence of small molecules (B-N) at different time intervals. Samples were removed at indicated times, snap frozen and stored at −80 °C for analysis on Native-PAGE. Concentration dependence of trehalose (B-C); sorbitol (D-F); Mannose (G-I); Serine (J-L) and TMAO (M-N) is shown. Lane 0 indicates pure AT protein, lane 1-9 indicates time 0, 5, 10, 15, 20, 45, 60, 75 and 90 minutes of incubation respectively.

**Figure S3:** Stoichiometries of thrombin (IIa) inhibition by AT in the absence and presence of small molecules.

**Figure S4:** Kinetics of polymer transition in the absence and presence of trehalose were assessed under polymerization conditions. Breifly, 100 μg/ml of native AT in a total of 1 ml was incubated at 60°C in PNE buffer, pH 7.4, in the absence and presence of trehalose. Samples were removed at indicated times and were assayed for thrombin progressive activity (in PNE-PEG buffer) to assess the loss of AT inhibitory activity due to transition to polymeric AT with time. Reaction for the measurements of activity was set up under pseudo first order condition and contained AT and thrombin in a 10:1 ratio. AT and thrombin were reacted in microplates, and following the Enzyme + Inhibitor incubations, S-2238 substrate was added and measured at 405 nm. Appropriate thrombin and S-2238 controls with trehalose in the absence of AT were taken.

**Figure S5:** Native PAGE Gel representing the heat induced polymerization of AT (2μM) in the presence of (A) 10mM Trehalose Octasulfate and (B) 50mM Trehalose Octasulfate

**Table S1:** List of small molecules used in in vitro screening.

**Table S2:** Secondary structure content of AT denatured with 2M GdnHCl in the absence and presence of 1M trehalose. Helical content was calculated as described [1].

**Table S3:** Alteration of secondary structure of AT in the presence of trehalose. Secondary structures were calculated using the online software k2D [1,2]

## Abbreviations

AT: antithrombin
Bis-ANS: 1,1′-bi(4-anilino)naphthalene-5,5′-disulfonic acid
GdnHCl: guanidine hydrochloride
MRE: Mean Residue Ellipticity
NEU: neuroserpin
PE: Phosphate-EDTA
PNE: Phosphate-NaCl-EDTA
RCL: reactive center loop
Serpin: serine protease inhibitors
SI: stoichiometry of inhibition
TEM: transmission electron microscope
Tm: melting point
TMAO: trimethylamine N-oxide
