# Supplementary material for: Deciphering the role of trehalose in hindering antithrombin polymerization
Source: Biosci Rep. 2019 Apr 5;39(4):BSR20182259. doi: 10.1042/BSR20182259 (PMC6449516; doi:10.1042/BSR20182259)
Supplement: Supplementary file 1 [file bsr-39-bsr20182259_Supp1.pdf]

SUPPLEMENTARY MATERIAL

Deciphering the role of trehalose in hindering antithrombin polymerization

Asma Naseem[a,*], Mohammad Sazzad Khan[a], Hashim Ali[b], Irshad Ahmad[a] and Mohamad Aman Jairajpuri[a,*]

[a] Protein Conformation and Enzymology Lab, Department of Biosciences, Jamia Millia Islamia (A Central University), New Delhi-110025, India

[b] Molecular Medicine Laboratory, International Centre for Genetic Engineering and Biotechnology (ICGEB), 34149 Trieste, Italy

[*] *Corresponding authors.*

Table S1: List of small molecules used in *in vitro* screening.

	Sugars and Polyols	CID No.	Chemical structure
1	Sorbitol	5780	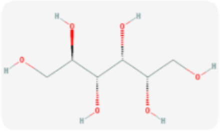
2	Mannose	18950	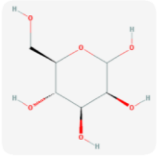
3	Trehalose	7427	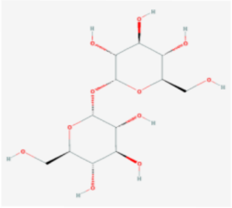
4	Maltose	439341	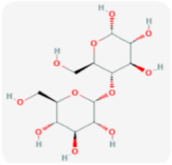
5	Dextrose	22814120	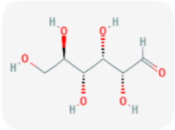
6	Galactose	6036	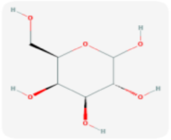
7	Inositol	892	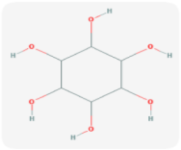
8	Mannitol	6251	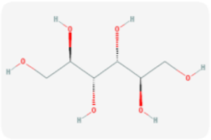

	Amino Acids	CID No.	Chemical structure
1	Alanine	5950	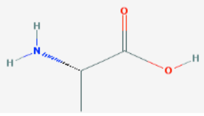
2	Glycine	750	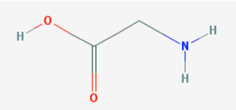
3	Lysine	5962	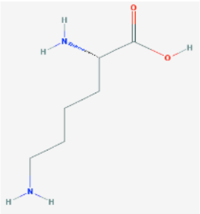
4	Arginine	6322	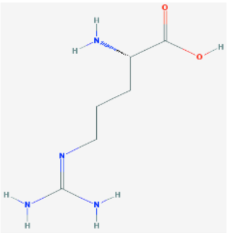
5	Serine	5951	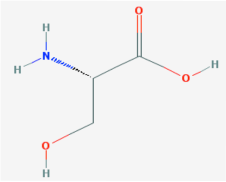
6	Threonine	6288	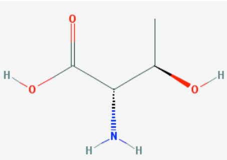
7	Ornithine	6262	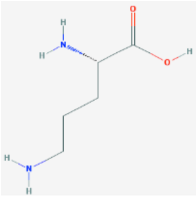
8	Citrulline	9750	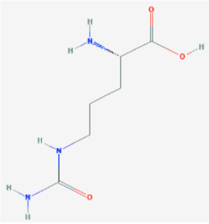
9	Cysteine	5862	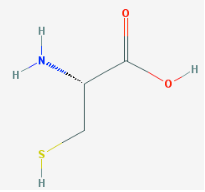

	Methylamines	CID No.	Chemical structure
1	Sarcosine	1088	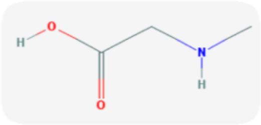
2	Betaine	247	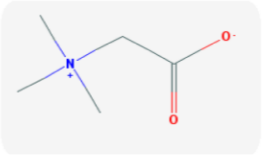
3	Trimethylamine N-oxide (TMAO)	1145	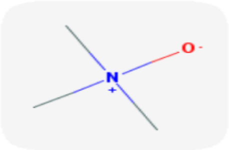

Table S2: Secondary structure content of AT denatured with 2M GdnHCl in the absence and presence of 1M trehalose. Helical content was calculated as described [1].

Molecule	MRE $_{222nm}$	%alpha helix
AT	- 4.3852	15.6 ± 0.03
AT+TRE	- 5.2993	21.7 ± 0.05

Table S3: Alteration of secondary structure of AT in the presence of trehalose. Secondary structures were calculated using the online software k2D [1,2]

Secondary Structure	AT	AT+TRE
α-helix (%)	29	36
β-sheets (%)	22	20
Random Coil (%)	49	43

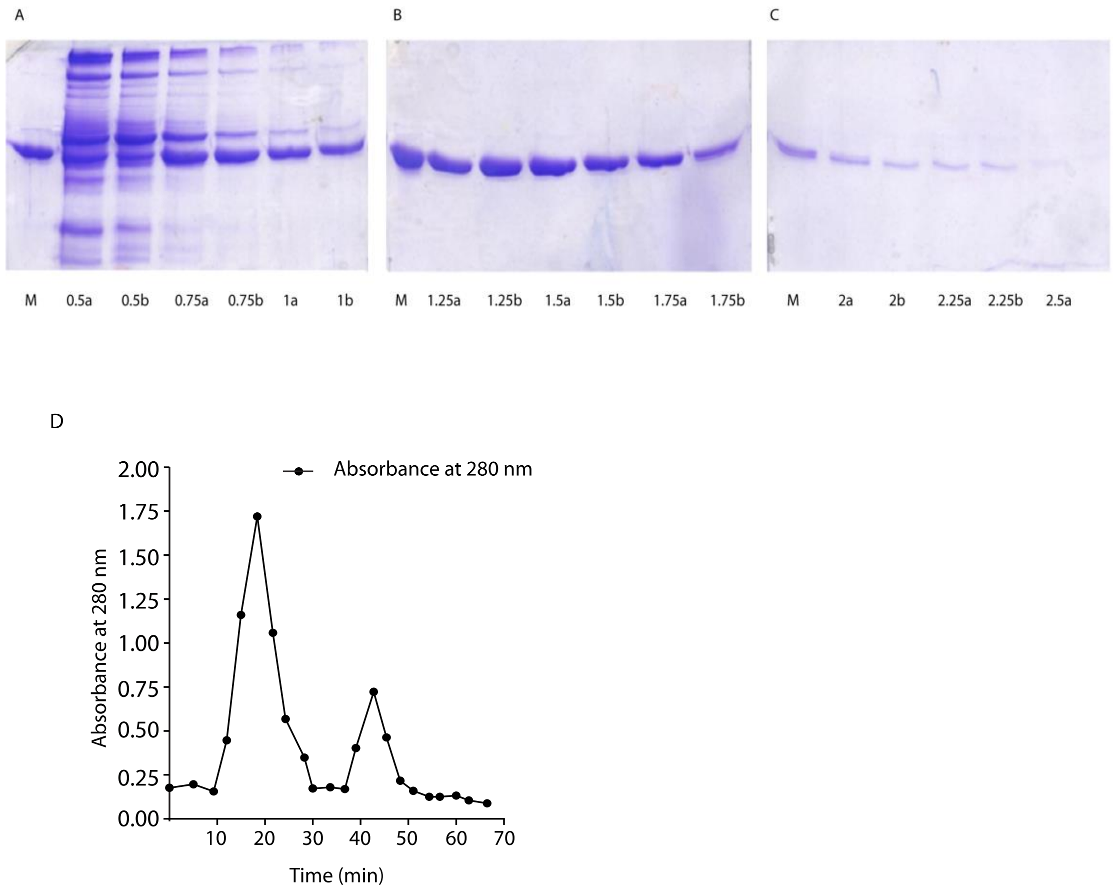

Figure S1: Purification of AT from human plasma. Elution profile of purified AT is shown. (A-C) SDS-PAGE of AT purified using a salt gradient of NaCl (20 mM phosphate buffer containing 100mM NaCl, 0.1mM EDTA, pH 7.4 and ionic strength 0.15). Elution of protein started from 0.15M NaCl upto 2.5M NaCl. (A) Lanes showing the fractions collected from 0.5M upto 1M NaCl gradient run in doublets as indicated. (B) Fractions collected from 1.25M upto 1.75M are shown as in A. (C) Fractions from 2M upto 2.5M NaCl are shown as in panels A and B. Fractions containing 0.15-0.5 M gradients were treated as wash. Fractions showing single bands of purified protein were pooled, desalted and used in the study. M denotes pure AT run as marker. Lanes denotes different concentrations of NaCl as indicated. (D) Absorbance profile of the eluted fractions is shown. Each fraction was eluted for 3 minutes and absorbance was read at 280nm.

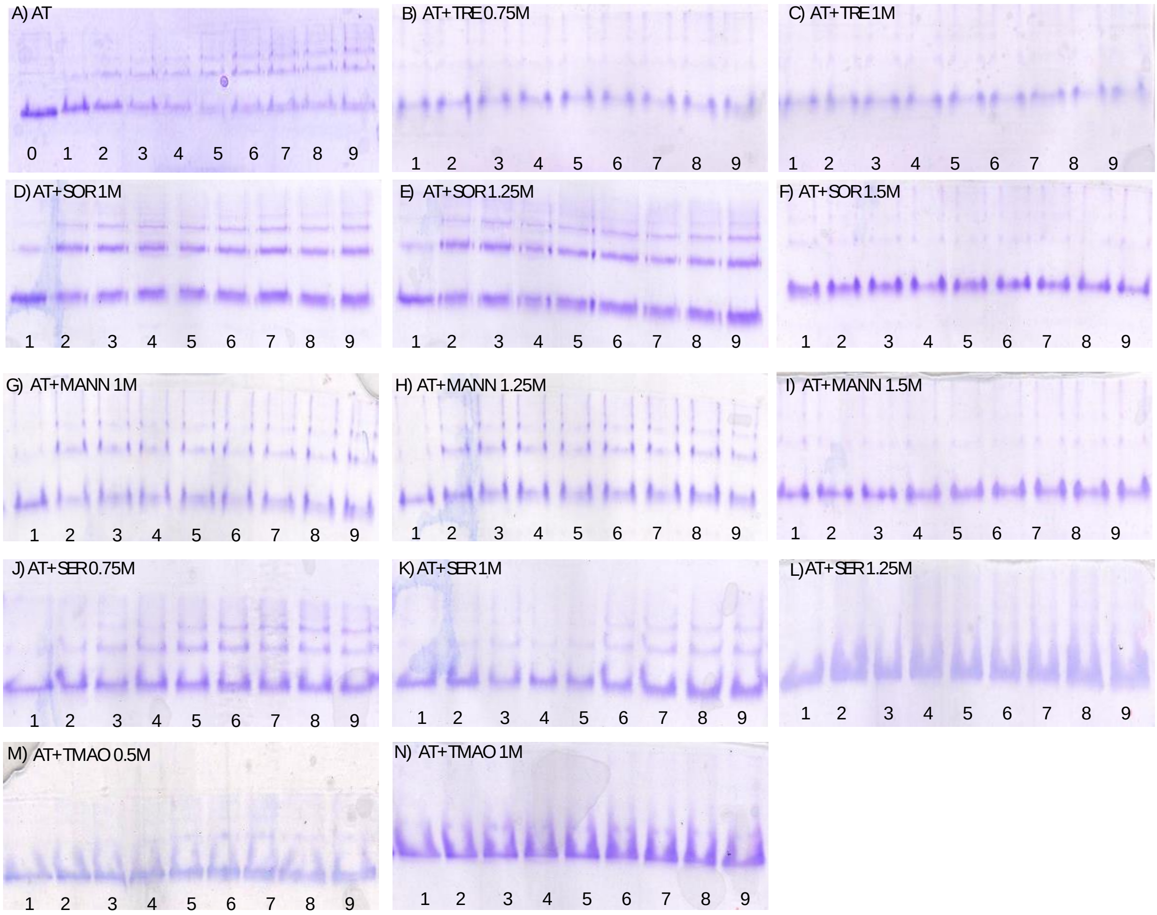

Figure S2: Concentration dependence of small molecules that successfully retarded AT polymerization. Polymers of AT were prepared by heating 100µg ml^{-1} of native AT in total of 1ml at 60°C in 50 mM Tris and 50 mM KCL buffer, pH 7.4 in the absence (A) and presence of small molecules (B-N) at different time intervals. Samples were removed at indicated times, snap frozen and stored at -80 °C for analysis on Native-PAGE. Concentration dependence of trehalose (B-C); sorbitol (D-F); Mannose (G-I); Serine (J-L) and TMAO (M-N) is shown. Lane 0 indicates pure AT protein, lane 1-9 indicates time 0, 5, 10, 15, 20, 45, 60, 75 and 90 minutes of incubation respectively.

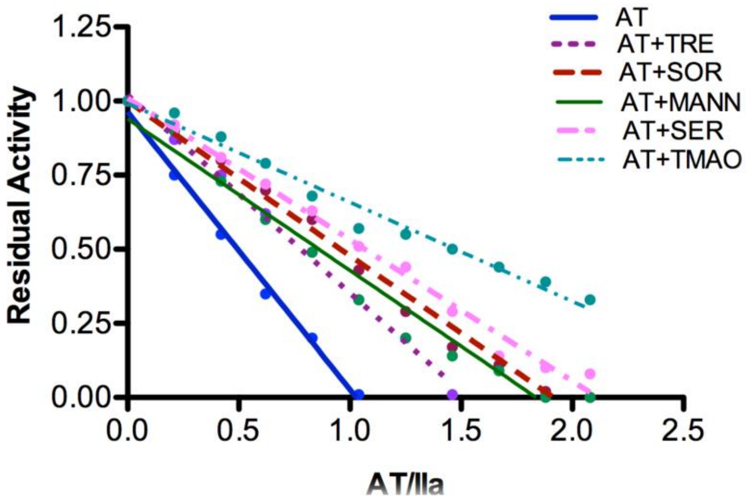

Figure S3: Stoichiometries of thrombin (IIa) inhibition by AT in the absence and presence of small molecules. Stoichiometries of thrombin inhibition were determined from residual protease activities with increasing AT concentrations as described previously (2). Briefly, 0–1000nM AT and 400nM of thrombin (IIa) in PNE-PEG buffer (20mM phosphate containing 100mM NaCl, 0.1mM EDTA and 0.1% polyethylene glycol 6000) were incubated at 25℃ in the absence and presence of 1M trehalose, 1.5M sorbitol, I.5M mannose, 1.25M serine and 1.25M TMAO. Residual enzyme activities were measured by adding 0.15 mM thrombin substrate S-2238 and recording the initial hydrolysis rate at 405 nm. SI's were taken as the x-intercept of the linear regression when residual protease activity is plotted against the ratio of AT to thrombin. Appropriate thrombin and S2238 controls/blanks with small molecules in the absence of protein were taken.

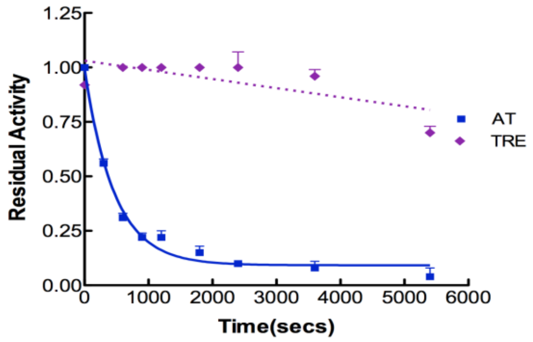

Figure S4: Kinetics of polymer transition in the absence and presence of trehalose were assessed under polymerization conditions. Breifly, 100 μg/ml of native AT in a total of 1 ml was incubated at 60°C in PNE buffer, pH 7.4, in the absence and presence of trehalose. Samples were removed at indicated times and were assayed for thrombin progressive activity (in PNE-PEG buffer) to assess the loss of AT inhibitory activity due to transition to polymeric AT with time. Reaction for the measurements of activity was set up under pseudo first order condition and contained AT and thrombin in a 10:1 ratio. AT and thrombin were reacted in microplates, and following the Enzyme + Inhibitor incubations, S-2238 substrate was added and measured at 405 nm. Appropriate thrombin and S-2238 controls with trehalose in the absence of AT were taken.

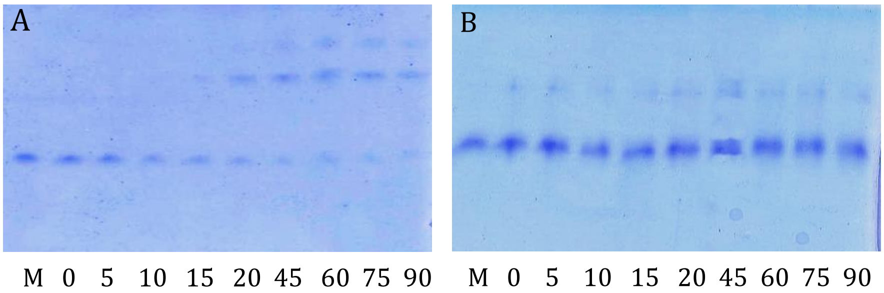

Figure S5: Native PAGE Gel representing the heat induced polymerization of AT (2µM) in the presence of (A) 10mM Trehalose Octasulfate and (B) 50mM Trehalose Octasulfate

Supplementary references:

[1] Crevenna AH, Naredi-Rainer N, Lamb DC, Wedlich-Söldner R, Dzubiella J. (2012) Effects of Hofmeister ions on the α-helical structure of proteins. *Biophysical Journal* **102(4):**907-15.

[2] Andrade, M. A., Chacón, P., Merelo, J.J., Moran, F. (1993). “Evaluation of secondary structure of proteins from UV circular dichroism spectra using an unsupervised learning neural network.” *Protein Eng* **6(4):** 383-890.

[3] Jairajpuri, M. A., Lu, A., Desai, U., Olson, S. T., Bjork, I., and Bock, S. C. (2003) Antithrombin III phenylalanines 122 and 121 contribute to its high affinity for heparin and its conformational activation. *The Journal of biological chemistry* **278**, 15941-15950
